# Introductory Lecture on Practical Anatomy for the Dentist

**Published:** 1854-01

**Authors:** W. R. Handy


### ARTICLE IX.

*Introductory Lecture on Practical Anatomy for the Dentist.* Delivered before the Class of the Baltimore College Dental Surgery—Session 1853-4. By W. R. HANDY M. D.

GENTLEMEN—Allow us to congratulate you on the present occasion—the occasion of entering upon a month's preparatory practical study. Your presence bespeaks your appreciation of this month's practical training, and we could only wish that more—yes, we might add all, would avail themselves of the practical advantages it offers.

We say this month is set apart as a preparatory month to the regular course of lectures—a month designed especially for practical study—a month in which you are to practice the different senses, to educate the *eye* and the *hand* particularly—a month, in which you are to gather together *facts* for future use, to lay a *foundation* upon which the after superstructure of your professional edifice is to be reared—a foundation, which to remain unshaken, must rest upon *science.* Yes, we repeat it, upon *science,* as the only legitimate basis, the only sure and trustworthy rock on which to build and practice the art of dentistry. All other foundations, we insist, are of sand, and will not stand the light of truth and investigation.

Every other art acknowledges science as its only *true* and proper basis. Why, we ask, should not the art of dentistry? There is, and can be no reason assigned why it should not. All the lovers and practitioners of this art, we mean those who sincerely desire its *elevation*, its *dignity* and *progression*, practice to this end. We say all such as these are practicing upon *science* as their foundation—are laboring hard, and making great sacrifices. Yes, we may safely say, not a few of such are at this day making great personal self-sacrifices, to lay still deeper, and wider, and stronger, and more enduring, the *foundations of dental science*. They acknowledge no other basis, either morally or pecuniarily, upon which as honest men, they can rest the hope of seeing their profession a respectable and useful one, and at the same time obtain and enjoy a conscientious, comfortable and satisfactory living.

Whilst on the other hand, all the rest who practice the dental art, (we refer to the self-styled dentists,) do not and will not care any thing about *science*—whose *name* is *legion*, and who practice every species of charlatanism under the guise of dental surgery, upon their unsuspecting and credulous patients, and with no other principle than that of making money, sacrificing every thing like *moral honesty*, *decency* and *knowledge*, and holding up as their only title to confidence, the recommendations of *ignorance*, *arrogance* and *boasting skill*.

This latter class, it is affirmed, have no *foundation of science* upon which to rest their loud pretensions, (and consequently no claim upon the community,) for either trust-worthy skill, or legitimate professional honesty.

*Science*, says one lexicographer, is "certainly grounded on demonstration"—says another, it is "art attained by precepts, or built on principles."

Art, then, it is perceived by these definitions, is *demonstrative science*, not demonstrative in the loose acceptation of the term, but demonstration "*grounded*" upon real and positive certainties—*grounded* upon *real*, *substantial* truths—upon *unerring principles* applied to art, and found to stand the actual test of practice.

An art, thus founded upon the certainty of demonstrative science, and resting upon the unalterable principles of truth, is a *safe, useful* and *ennobling art.* And every art not so founded, we may as confidently affirm, is not safe, not useful, not ennobling, but, on the contrary, hap-hazard in its skill, highly injurious in the vast majority of cases, and positively destructive in a great many others.

*Dental art,* gentlemen, which you have chosen for your profession, does not form an exception, but like all the rest, must also be based upon the firm foundations of science—which is called dental science. Your art must also be studied, as industriously studied, by all the lights of said science, as any other art. And still further, by this same science each of you must expect to practice, habitually practice, the dental art, if you would wish to have it, as it always should be, a safe, useful, ennobling and respectable art.

What, it may here be asked, is dental science? What are its foundations? What its limits? What its requirements, necessary to be fulfilled before one is competent to undertake and practice the dental art?

We reply that *medical science, chemical science,* and *mechanical science* constitute the *fundamental* and *essential elements* of *dental science.* These are the *foundations* necessary to be laid, before you can expect to practice dentistry safely, successfully and conscientiously.

*Practical Anatomy,* is one of the branches of medical science, and thus forms part of one of the foundations stated as necessary to dental science. And as it is this portion which belongs most especially to our department, and of which it becomes us the more properly to speak, we shall, therefore, confine our remarks very briefly to this one point, leaving the rest of the fundamental elements of dental science to be explained by the appropriate chairs for this purpose.

What, then, are we to understand by *practical anatomy* for the dentist? It is hardly necessary to reply, gentlemen, that we mean the *anatomy of the knife—the anatomy of real, actual dissection—anatomy* requiring the diligent and persevering use of the *eye* and the *hand*, and not confined to book anatomy, and the anatomy of the plates, acquired by memory. Such anatomy as these latter furnish, is not practical anatomy—is not the kind of anatomy which will help you in time of need, when you each shall engage in practice. The anatomy of the books and plates are very necessary and useful, but only so far as helps in the actual dissection of the body itself, in showing you how to display its several parts, and never designed to supersede the knife. But some may be ready to ask, as it has been asked, what has *dissection of the human body* to do with the practice of dentistry, or at most any further than the mouth is concerned? The answer to this question, involves the most we have to say in reference to practical anatomy. And in reply, the *first remark* we have to offer is, that *dentistry* cannot be a *science* without the *aid* of *practical anatomy*.

This position requires very little proof, if the definitions already given of science, be received as conclusive. For if medical science has, as universally admitted, practical anatomy for its basis, and if dentistry is only a branch of medicine, then it necessarily follows that practical anatomy is, also, the basis of dental science. We, therefore, pass on to our *second remark*, which is a necessary and inevitable consequence of the first, viz. that dentistry, *as an art*, cannot be practiced properly, that is in accordance with science, without the aid of practical anatomy.

If this position, gentlemen, be true, it is all important that you should know it; and know it, not only as a common, vague and general truth, but know it experimentally, as a *great practical principle*, to be tested by each of you individually, if you expect to attain the professional standing of scientific dentists. As this is a point of much practical interest to each of you, it may be necessary to offer here a brief explanation.

How then is dissection to aid you in the practice of dentistry? If such a question were seriously put to a student of medicine or surgery, it would occasion the greatest surprise; even the slightest intimation that dissections could be dispensed with, would be regarded as showing either great stupidity or ignorance. Now why should there be this difference of feeling between the student of medicine and surgery, and the student of dentistry? We feel safe in replying there is and can be no reason for the difference, for the same line of argument that establishes the necessity of dissections in the first case, applies with equal force to the second.

To show that such is the fact, our proposed explanation on this head, will be best given by running a brief contrast between the two cases just mentioned, viz. of the student of medicine on the one hand, and the student of dentistry on the other.

The student of medicine and surgery urges that the practice of his profession deals exclusively with the human body, with all the organs composing this body collectively, as well as each individual organ, considered separately, and that to attempt to restore such a body when injured as a whole, or in any of its parts, without an acquaintance from previous dissections, would be equally if not more absurd and presumptuous than for an individual to attempt the repair and regulation of a watch, about which he knows nothing.

It is further urged, and very justly, that it is not only necessary to know that there are organs composing the human body, but further, and what is more important, that it is absolutely necessary to know where these organs are, what is their natural location, what their healthy form, size, color, consistency, structure and relations.

Now it is contended, that all such knowledge as this can only be obtained by means of *actual*, *individual* dissections, such dissections as require that each student for himself, shall lay hold of the knife, and faithfully and perseveringly use it, in the exploration of the wonderful mechanism of man's organization; in other words, that there can be no *proxy* in the matter, but on the contrary that the *eye* and the *hand* of each must be thoroughly drilled, and educated, to see and know for himself; for every deviation from the natural, healthy, physiological condition of an organ, whether that deviation be in the location, form, size, color, consistency or structure, constitutes in the same proportion disease, and this disease or deviation being only cognizable by the eye and the hand, must, of necessity, have these senses practically exercised to be able to detect it.

It is true, the books and plates will accurately tell us where organs are, what their form, color and all the physical characteristics belonging to each; but this is not practical knowledge, the knowledge which is to avail you when you are brought to the bedside of disease, or have to operate. Your knowledge then to be applicable and trust-worthy, must be such as you each do know, and have tested for yourselves. What, suppose you, would be thought of that surgeon who would attempt to tie the carotid or femoral artery, or perform the operation of hernia, without ever having seen or handled the parts involved? Why, the most liberal judgment, in view of such utter recklessness of human life, could not pronounce such surgeon any thing less than a madman. Or what would be thought of that physician who could talk learnedly from the books, of congestion, hepatization, dropsy, tubercles, pleurisy and other affection of the chest, who would boast, at the same time, of his superior skill in their treatment, and yet have never seen the lungs, the windpipe or the pleura? Or what would be thought of either surgeon or physician, who would mistake ague and fever and give bark and quinine, when such ague simply depended on the sympathetic relation between the urethra and spinal marrow, produced by the introduction of the catheter? And so the same remarks apply to each and all the organs of the body, showing that dissections are indispensable to properly qualify one for practice.

Now how is the case with the student of dentistry? Has he not to deal with the human body and its different organs, as well as the medical and surgical student? Does he not also have to inquire into disease and its cause? Can he escape, without deserved censure, the equal necessity of tracing the relations of the several organs? If an affirmative reply be given, the necessity of dissections must be admitted.

It may be replied, however, that the dentist has not to do with the whole body—that his duties are strictly confined to the teeth and its diseases, and that he is travelling out of his sphere when he goes beyond that. But diseases of the teeth, especially their sympathetic diseases, depend, for their cause, on disease in some one or more remote organ or organs. As a skillful dentist and a master-workman in your art, would you be justified at the bar of conscience and before your patients, in pleading as an excuse for sacrificing their teeth, that the cause of the mischief was in some other organ and not in the teeth, as you had suspected; that you were sorry for the mistake, but as you had nothing to do with any other part of the body but the teeth, of course there was no blame to attach to you in the matter?

Would any of you gentlemen, for one moment, attempt to offer such an apology? No! We would venture the assertion, there is not a man, who is not profoundly ignorant or knavish, that would not be ashamed (and so ashamed as not even dare) to offer such an excuse in the presence of deceived and suffering humanity.

Your art embraces the duties of both physician and surgeon, and your science claims for its basis the firm foundations of both medical and surgical science.

The teeth, you all know, are organs—that as other organs they have blood-vessels and nerves—that by these latter they are connected with the rest of the system, as other organs—that this relationship is as strong for weal or woe (in proportion of the teeth's vitality) as that of any other part of the body, and that, as the rest of the organs, they also grow, attain maturity, become diseased, decay and die. In a word, health, disease, decay and death, attaches to the teeth equally with all other organs, and in the management of their diseases require an equal amount of skill and knowledge.

We will briefly enumerate a few of the more prominent diseases of other organs arising from diseases of the teeth, and disease of the teeth from disease in other, and often distant organs, each alternately becoming *cause and effect*, so as to impress upon your minds the *natural* and *inseparable relation of the teeth* with *each* and all the *organs* composing the system, and still further to impress the other fact, that so close is this relation that it is impossible to practice successfully in either without a knowledge of both.

*Convulsions*, *photophobia*, *otitis*, *hydrocephalus* and *dys-*

*pepsia*, constitute a few of the affections arising from disease of the teeth. Convulsions occur during dentition. The fifth pair of nerves are the great sentient nerves of the dental apparatus, and its superior and inferior maxillary branches, especially, supply the teeth. It is by these latter branches that the painful impression of dentition is carried to the brain, and through the brain, by means of the motor nerves, acting on the voluntary muscles, it is, that this frightful muscular disturbance, called convulsions, is produced. Photophobia is a dread of light, as its name implies, and consists of excessive, and the most painful sensibility to the impressions of light. The first branch of opthalmic division of this same fifth pair of nerves goes to the eye, and readily explains the medium of production of this terrible affection, when the teeth are the cause.

The American Journal of Medical Science records a most interesting case of this disease, where, after every kind of treatment had been tried, nothing but the extraction of a few teeth did any good; and this, the record informs us, most promptly and effectually cured the affection, showing most conclusively that the teeth were in fault, and that they were the cause of all the mischief. This case teaches a most useful lesson to physicians, who are so prone to overlook or even to suspect the teeth as being the sources of mischief beyond the limits of the mouth.

Otitis, hydrocephalus and dyspepsia, when originating from disease of the teeth, may be explained to be produced through the medium of the fifth, associating with the eighth and sympathetic nerves.

Disease of the antrum or maxillary sinus, whether it be inflammation, suppuration or tumor in this cavity, does not require any special explanation as to the manner of origin, for the antrum and teeth being close together—so close, indeed, that the latter are not unfrequently found to penetrate the former—readily accounts for disease of the one passing into that of the other, solely, as physiologists express it, by the sympathy of contiguity.

If we now turn to the second class, we here find in the stomach, salivary glands, uterus, extremities and organs of circulation, examples of disease of other organs acting upon and producing disease in the teeth. When the stomach is surcharged with acidity, and the salivary glands have their fluids vitiated, the teeth are liable to decomposition from the action of these altered agents. Disease of the uterus, especially during pregnancy, may cause the most violent pain in the teeth, and injuries of the feet have often locked the jaws with that most formidable disease, termed trismus or locked-jaw, a spasmodic affection so violent that the teeth cannot be separated, so as to open the mouth, without resort to great force. These are all sympathetic diseases of the teeth that are dependent upon, and have their origin in some one organ or organs more or less remote. They bear, in their symptoms, all the semblance of reality; so much so, indeed, as sometimes to baffle the most scientific in their detection. These sympathetic diseases, however, of the teeth, should, and must be detected, if you wish to save yourselves the mortification of extracting sound ones, and of incurring the censure of ignorance and a want of skill in the practice of your profession.

But, gentlemen, we respectfully ask, how are you to escape such censure if you scarcely know anything of the existence of such organs—nothing at all of their locality—and still less of the relations of these organs with the teeth, upon which relations depend the sympathetic diseases of the latter? The *cause* of these sympathetic disorders of the teeth you would never be led to suspect of being in some other part of the system, if you confine your attention solely to the teeth, and look no further. And if, with all the light of science, and the most persevering and thorough *dissections* of the whole body, the greatest skill has been baffled, how can those expect to succeed who totally neglect dissections, or so far deny their utility as to think that the smallest quantity possible will suffice for all practical purposes.

This latter class are willing to concede, that it would be as well, perhaps, to dissect the mouth—not, however, with any high and ennobling motives for the discovery of facts and principles by which to attain to a more skillful and successful practice. Not at all; but, on the contrary, from the operation of that lower and baser motive—that it would not look genteel in fashionable society—not to know anything about the mouth; and still further, if something was not known, at least in a general way about it, so that a *quantum sufficit* of learned nonsense should be at hand for all occasions, why nothing would be gained by it.

This remark, gentlemen, may appear as if bordering too much upon an extreme, amounting, it may be thought, to an unjustifiable caricature of the matter. Allow us to reply, that if you do not know it now, you will hereafter, very soon, find it to be sober, undoubted reality. For how many, we may ask, of those self-constituted dentists, who only pretend to an acquaintance with the mouth, and who ignore a knowledge of all the other parts of the body as totally useless. How many, we ask, of such as these, can even tell you where the *palatine artery* is, of which, there is a constant liability to injury in the daily operation of lancing the gums? Or how many of them know where the *superior constrictor of the pharynx* is situated, a muscle, which is so near the posterior teeth of the lower jaw, as to readily incur the risk of being divided, and the consequence of which injury would be the inevitable crippling of the function of deglutition, as well as serious impairment in the use of the voice?

The answer would, most undoubtedly be, that few, if any of such dentists were aware, or even suspected that such dangers were constantly in their path, and ever present in all their daily operations on the mouth; and yet they will lay claim to a knowledge of the mouth, proving most conclusively that they have not even a book-knowledge, much less a practical knowledge from *dissections*.

In *conclusion*, gentlemen, we trust enough has been said to show that the *art of dentistry* is based upon *principles—scientific principles*—resting upon the *firm foundations* of demonstrative truth, and as a part of which truth, *practical anatomy*—the anatomy of dissections—forms an *essential* and *indispensable element* in enabling you to conscientiously and successfully practice this art.

